# *In-situ* preparation of functionalized molecular sieve material and a methodology to remove template

**DOI:** 10.1038/srep22813

**Published:** 2016-03-10

**Authors:** Rekha Yadav, Maqsood Ahmed, Arvind Kumar Singh, Ayyamperumal Sakthivel

**Affiliations:** 1 Inorganic Materials & Catalysis Laboratory, Department of Chemistry, University of Delhi, Delhi 110 007, India

## Abstract

A series of diaminosilane-functionalized silicoaluminophosphate molecular sieve (SAPO-37) was prepared by *in-situ* synthesis, and a novel method was developed for the selective removal of structure directing agent (SDA)/template from the functionalized SAPO-37.The complete removal of the SDA was evident according to FT-IR, TGA, ^13^C MAS-NMR and elemental analysis. The developed method was found to be efficient for removal of template from microporous molecular sieve viz., SAPO-37 and can be applied for other microporous molecular sieves such as SAPO-5, SAPO-40, etc. The powder XRD pattern of the template-removed samples showed a highly crystalline SAPO-37 phase. Argentometric titration revealed that more than 90% of diamine functionality exposed on the surface was accessible for catalytic applications. The resultant materials showed promising activity for ring opening of epoxide with aniline to yield β-amino-alcohol.

The synthesis of zeolite and zeolite like microporous molecular sieves with organic functional groups within the channels and framework can tailor their textural as well as catalytic properties depending on the nature of the functional groups incorporated^1^,^2^,^3^,^4^,^5^,^6^,^7^,^8^,^9^,^10^,^11^. In general, organic functional groups have been introduced on the surface of molecular sieve materials either through *in-situ* co-condensation using organosilane precursors or by post-synthetic functionalization. The *in-situ* introduction of organic functional group in inorganic framework, results in organic-inorganic hybrid materials possessing unique properties such as hydrophobic environment, adsorption capacity etc. The hybrid materials are synthesized in hydrothermal conditions and have organic group either as pendent hung within pore or partially substituted either inside the framework or on external surface^12^,^13^,^14^,^15^. The post-synthetic grafting method has been studied extensively for mesoporous materials which are applied in various applications, such as CO_2_ capture^16^,^17^,^18^ and base-catalyzed reactions^19^,^20^,^21^,^22^,^23^,^24^. Though mesoporous molecular sieves based materials shown promising applications, their commercial applications are limited owing to poor stability and amorphous wall properties^25^. Although, post-synthetic method covalently anchors functional groups on the surface of molecular sieve materials increasing in surface functionality, it is difficult to control the distribution of functional groups which results in non-homogeneous allocation of functional groups on surface and within the channels^26^.

Faujasite (FAU) type microporous molecular sieve possessing 3-D pore structure with pore opening of 12-membered ring and large sodalite cages are commercially important in crude oil industries (about 40% conversions)^27^. Further introduction of organic functionality in microporous molecular sieves will result in new class of shape selective catalysts with organic active group. Three-dimensional framework materials with organic groups were first reported by Maeda *et al.*^1^ by *in-situ* hydrothermal synthesis of an aluminophosphate molecular sieve with methyl functionality. Aluminophosphate materials are important owing to the flexible framework where different metal ions can be incorporated to tune the acidity^28^,^29^. Subsequently, a series of molecular sieves containing different functional groups have been synthesized, and they have been proven important for hydrocarbon sorption and several other applications^1^,^2^,^3^,^4^,^5^,^6^,^7^,^8^,^9^,^10^,^11^,^19^,^20^,^21^,^22^,^23^. For microporous molecular sieve materials, the co-condensation method can facilitates high-loading of functional groups by accommodating the organosilane into the framework^24^ or complete distorted products due to disturbance in the crystal growth. In particular, for silico-aluminophosphate (SAPO) based materials, there are only limited reports on functionalization^7^,^15^,^16^,^17^,^18^. In this regard, it is emphasized that faujasite type zeolites showed immense potential for petrochemical processes. The analogous faujasite type silicoaluminophosphate (SAPO-37) has limitations as it is known that the structure collapse while removal of tetramethylammonium hydroxide (TMAOH) and exposure to water. It would be interesting to introduce amine functionality in faujasite type silicoaluminophosphate (SAPO) molecular sieve using *in-situ* hydrothermal synthesis in a controlled manner.

Unlike functionalized mesoporous silica, the applications of functionalized microporous materials are limited owing to the difficulty in selective removal of structural directing agent (SDA) from microporous molecular sieves^7^,^30^. In general, removal of structure-directing agents (SDAs) from functionalized mesoporous materials are carried out either by solvent extraction or by ion-exchange method^31^. In case of microporous materials, SDAs removal is a challenging process because of strong interaction of inorganic precursors with SDAs^30^. The solvent extraction of SDAs for microporous materials, and treatment with chemicals such as ethanolic HCl and acetic acid damages the framework structure of microporous materials causing dealumination. It is essential to develop a SDA extraction route for the functionalized microporous materials without using conventional mineral acid viz. HCl.

To the best of our knowledge, there have been no report on *in-situ* introduction of diamine functionality in FAU type SAPO-37 framework as well as the complete removal of SDAs from functionalized microporous molecular sieves. Here, we report for the first time *in-situ* synthesis of diaminosilane-functionalized faujasite-type silicoaluminophosphate (SAPO-37-DAS), and describe a novel method for the removal of SDAs by ion-exchange using ethanolic sodium nitrate solution (supplementary Figure S1). This is unique method on removal of a SDA using ion-exchange method for microporous molecular sieve materials.Materials synthesized with different concentrations of N-[3-(trimethoxysilyl)propyl]ethylenediamine (DAS) 0.04, 0.08, 0.12, 0.16, 0.24 and 0.32 are labeled as SAPO-37-DAS-0.04, SAPO-37-DAS-0.08, SAPO-37-DAS-0.12, SAPO-37-DAS-0.16, SAPO-37-DAS-0.24 and SAPO-37-DAS-0.32 respectively (Table S1).The resultant SDA-extracted materials (represented by prefix ext) ext-SAPO-37-DAS was utilized as base catalyst for the ring opening of epoxide using aniline.

## Results and Discussion

Both as-prepared and template extracted diaminosilane SAPO-37 were white in color, with no other aggregated phase, indicates the successful incorporation of diamine-functionality in the framework of SAPO-37. The success of *in-situ* synthesized diaminosilane functionalized SAPO-37 could be its synthesis conditions involving pH = 7–7.8 in which rate of hydrolysis of organosilane is slow. Thus organosilane did not condense to form an aggregated phase but rather uniformly condensed within the inorganic species of the framework homogeneously^32^.

Figure S2 shows the powder X-ray diffraction patterns of as-prepared diaminosilane functionalized SAPO-37. The diffraction patterns of all the samples (SAPO-37-DAS-0.04 to SAPO-37-DAS-0.32) exhibited characteristic reflections corresponding to the SAPO-37 framework^33^,^34^,^35^. The XRD patterns remained intact with a highly crystalline phase even in the presence of large amount of diaminosilane functionality introduced, suggesting that the faujasite-type SAPO-37 framework structure was preserved during synthesis. The above fact was further confirmed through Le Bail fitting method, XRD patterns of all the materials are fitted (Le Bail method) using Fd-3m space group allowing determination of respective unit cell parameters and confirming the purity of FAU material obtained (Figure S3). A very weak XRD reflection was evident at a 2θ value around 18 for the higher-diaminosilane loaded sample (SAPO-37-DAS-0.32), which is characteristic of alumina impurity and could have resulted due to replacement of aluminum in the framework by organosilane functionality. Additional weak 2θ peaks near 7 and 14° were evident at low silane concentrations (in the range of 0.04 to 0.08) were assigned to AFI phase. Because the silica concentration is crucial in the formation of SAPO-37 with a faujasite structure, the samples obtained using 0.12, 0.16 and 0.24 M of diaminosilane in initial gel composition led to pure SAPO-37 phase.

**Figure 1:**
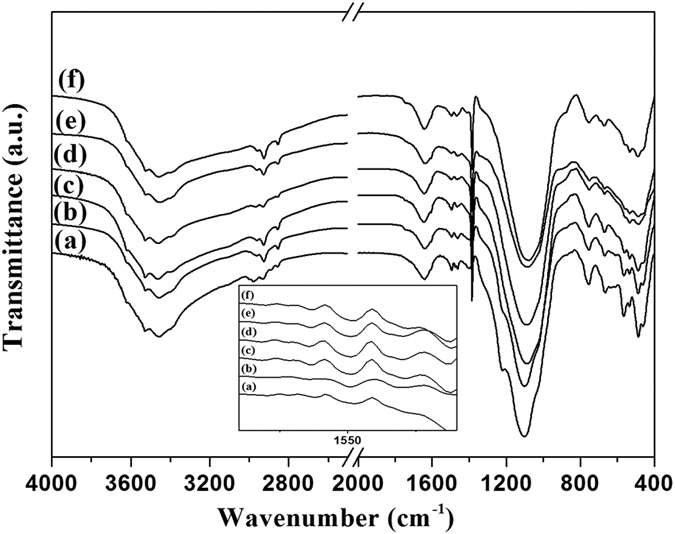
FT-IR spectra of extracted diaminosilane functionalized SAPO-37 with organosilane concentration (**a**) 0.04 (**b**) 0.08 (**c**) 0.12 (**d**) 0.16 (**e**) 0.24 and (**f**) 0.32.

The FT-IR spectra for as-synthesized SAPO-37-DAS-x (where x = 0.04, 0.08, 0.12, 0.16, 0.24, and 0.32) are shown in Figure S4. The bands at 561 cm^−1^ correspond to the double six member (D6R) of the secondary building units (SBU) of faujasite-type SAPO-37. The additional band at 535 cm^−1^ can be attributed to phosphorous in the D6R unit of AlPO framework. The broad band in the region of 1108 cm^−1^ corresponds to the asymmetric stretching frequency of T-O-T (where T = Si, Al and P). The absorbance bands in region 3500-3650 cm^−1^ can be assigned to hydroxyl group of either surface or physisorbed water molecules present in super-cage and sodalite cage of SAPO-37^33^. The relatively broad peak at 1493 cm^−1^ and 1560 cm^−1^ (shown in inset) are assigned to the bending mode of NH and NH_2_ functionality derived from different amines present in the framework of SAPO-37. The peaks in the range of 2700–3000 cm^−1^ appear due to stretching frequency of CH derived from various environments, such as template, organo-functional silane, etc. In the present studies, a novel method was utilized to remove the template (tetrapropylammonium (TPA^+^) and tetramethyl ammonium (TMA^+^) ions) by ion-exchange method using ethanolic sodium nitrate solution. Figure 1 shows the FT-IR spectra of samples after extraction. The peaks around 2700–3000 cm^−1^ corresponding to the stretching vibrations of the CH functionality present in the SDA are drastically decreased. The peak intensity corresponding to the NH vibration around 1560 cm^−1^ remains intact, which support the removal of template/SDA from SAPO-37 faujasite framework by ion-exchange with ethanolic sodium nitrate without disturbing the functionality. However the vibration around 1560 cm^−1^ remained masked in as-synthesized materials due to presence of higher concentration of template. The reduction in carbon content of the ext-SAPO-37-DAS-0.16 is shown in Figure S5. For further confirmation, FT-IR studies on as-prepared and extracted samples were performed after degassing at different temperatures under vacuum, and the results clearly showed (not shown here) that most of organic SDA were removed after washing.

**Figure 2:**
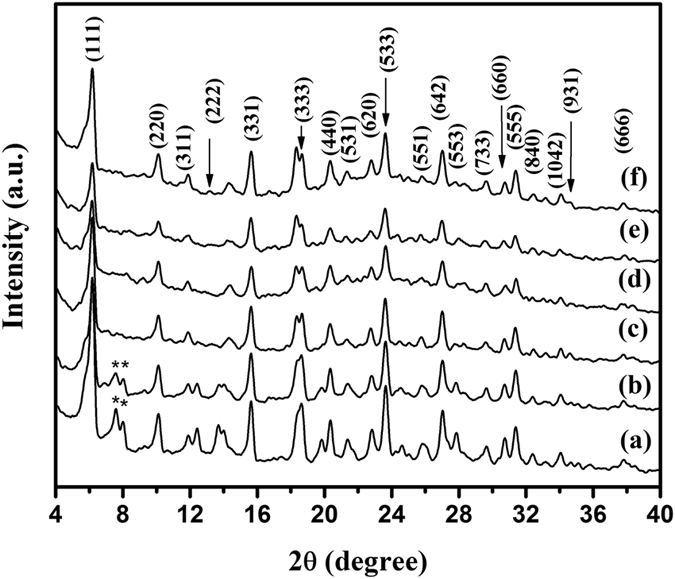
Powder XRD pattern of extracted diaminosilane functionalized SAPO-37 with organosilane concentration (**a**) 0.04 (**b**) 0.08 (**c**) 0.12 (**d**) 0.16 (**e**) 0.24 and (**f**) 0.32.

The powder XRD patterns of extracted samples (Fig. 2) retain all the reflection characteristics of SAPO-37 phase with relative broadening of the peaks owing to surface roughness caused by the presence of diamine functionality on the surface. The crystalline sizes of the as-prepared and extracted crystallites based on pattern fitting using Le Bail method showed Fd-3m space group for all diaminosilane functionalized SAPO-37 materials. Further the unit cell parameters were calculated using the above method are shown in Table S2 (a = b = c ≈ 27.7 Å) confirming the purity of faujasite type SAPO-37 phase. The refinement data clearly demonstrates that there is no appreciable change in unit cell parameters after the extraction of template by ion-exchange method, which shows that the current extraction method is efficient to remove SDA without damaging the SAPO-37 crystallite framework.

**Figure 3:**
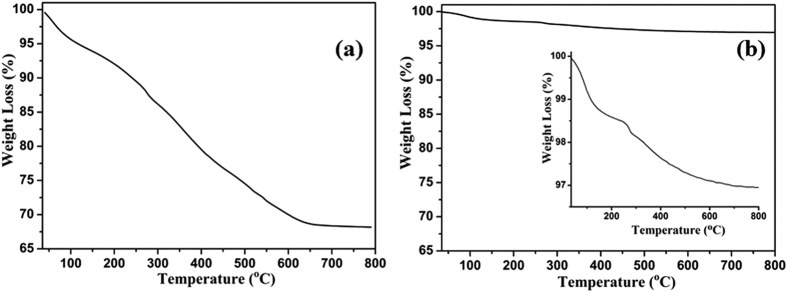
TGA of SAPO-37-DAS-0.16 (**a**) as-prepared and (**b**) extracted material.

In order to confirm the effective extraction of template from the microporous channels of SAPO-37, TGA of as-synthesized and extracted SAPO-37-DAS-0.16 was performed in air in a temperature range of 25 to 800 °C, and the results are displayed in Fig. 3. The as-prepared SAPO-37-DAS-0.16 sample (Fig. 3a) showed a total weight loss of about 33% accomplished in a three-step weight loss. (i) The first step weight loss corresponds to physisorbed water (up to 120 °C). (ii) The second weight loss (17 wt.%) in the temperature range of 200-400 °C was due to SDA (TPA^+^) present in the super-cage of SAPO-37 along with organosilane functionality. (iii) An additional weight loss of about 10 wt.% was due to loss of SDA (TMA^+^) from the sodalite cages of SAPO-37.

The template-extracted SAPO-37-DAS-0.16 sample showed a total weight loss of only 2–4 wt.% (Fig. 3b) in the temperature range of 200–400 °C (inset), which was due to organosilane moiety covalently bonded in the framework of SAPO-37, which is in very good agreement with FT-IR studies. Furthermore, an additional weight loss of about 0.5 wt.% in the temperature region of 400–700 °C could have derived from trace residuals SDA (TMA^+^) present in the sodalite cage. This study clearly shows that sodium nitrate extraction method efficiently removes the template from diaminosilane functionalized SAPO-37. The extraction method have been extended to MESO-SAPO-37 (as shown in Figure S6) prepared as per the procedure described earlier^34^,^35^, and other microporous systems viz. SAPO-40, SAPO-5 (as shown in Figure S6). The results suggest that, in both microporous (SAPO-40 and SAPO-5) and mesoporous molecular sieve (MESO-SAPO-37) complete removal of template was evident (Figure S6). Above results suggest that ethanolic sodium nitrate method efficiently works for removal of template from microporous materials.

Further, the efficiency of present template extraction method was compared with various conventional methods such as HCl-ethanol, acetic acid-ethanol and NaCl-ethanol using SAPO-37-DAS-0.16.The powder XRD profile and FT-IR spectra of SAPO-37-DAS-0.16 after template extraction using different methods are shown in Figure S7. It is clear from the XRD profile that SAPO-37-DAS-0.16 extracted using ethanolic NaNO_3_, NaCl, and acetic acid retained all the reflections characteristic of SAPO-37 phase. The use of ethanolic sodium chloride and acetic acid for template extraction result an addition peak around 2θ of 18, which is characteristic of alumina that could have resulted due to aluminum leaching from the framework. Further, ethanolic NaCl extracted sample showed another new peak around 2θ of 31.7 derived from impure phase developed. However, the sample extracted using ethanolic HCl showed adverse effect on SAPO-37 structure and most of the SAPO-37 phases were collapsed. It is well known that SAPO-*n* systems are not stable in acidic conditions, the use of ethanolic HCl not only remove the template, it also removed hetero atom from microporous framework, which facilitates the structure collapse. The above results support that conventional ethanolic HCl method which is used for removal of surfactant on pure silica based systems may not be suitable for aluminosilicates and silicoaluminophosphates systems. The FT-IR spectra (Figure S7B) further support that the sample obtained after template extraction using ethanolic HCl, does not show vibrational bands corresponding to SBU of FAU structure due to loss of SAPO-37 phase. Although ethanolic acetic acid and sodium chloride showed partial removal of template from microporous SAPO-37 framework, considerable amount of alumina leaching was also evident. Hence, the method developed in the present study, i.e. ethanolic sodium nitrate has ability for selective removal of template without damaging the microporous SAPO-37 framework. Thus, for the further studies, the SAPO-37 DAS materials extracted using ethanolic sodium nitrate was used.

**Figure 4:**
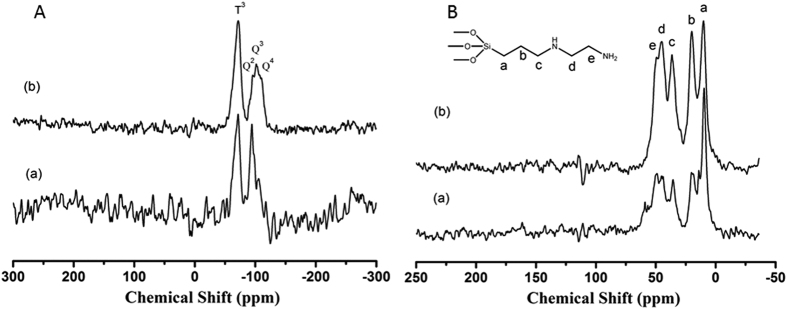
Solid state MAS NMR (**A**) ^29^Si and (**B**) ^13^C of SAPO-37-DAS-0.16 (**a**) as-prepared and (**b**) extracted material.

The chemical environment of the diaminosilane functionalized SAPO-37 materials was derived from ^29^Si and ^13^C MAS NMR studies. The ^29^Si and ^13^C MAS NMR spectra of as-prepared and template extracted SAPO-37-DAS-0.16 material are shown in Fig. 4. The ^29^Si MAS NMR spectrum (Fig. 4A) of both samples revealed the presence of T and Q sites characteristic of organosilane and inorganic silane environments, and it is expected that the incorporation of Si in place of P resulted in the formation of Si(4Al)^32^,^33^,^36^,^37^,^38^. The as-prepared SAPO-37-DAS-0.16 sample shows presence of broad resonance peaks at **−**71 and **−**94 ppm with a small hump at **−**105 ppm corresponding to different silicon environments. The peaks at −94 and **−**105** **ppm correspond to Si(3Al,Si) and Si(0Al,4Si), respectively^38^. The peak at −71 ppm was attributed to organosilane (T^3^) sites resulting from the complete condensation of diamino-propylsilane groups in SAPO-37 framework. This confirms the covalent incorporation of silane moieties in the framework of SAPO-37, which is in agreement with the results of FT-IR and TGA studies.

The ext-SAPO-37-DAS-0.16 showed peaks at −71, −89, −96, −101 and **−**110 ppm corresponding to T^3^, Si(4Al), Si(3Al,1Si), Si(2Al,2Si) and Si(0Al,4Si), respectively (Fig. 4A). The extracted materials showed more Si(2Al,2Si) sites than as-synthesized materials. According to the literature, presence of flexible framework on aluminophosphate molecular sieve allows substitution of different hetro-atoms in the framework. In faujasite materials, substitution mechanism 3 is prominent which involves replacement of both Al^3+^ and P^5+^ from consecutive sites for Si substitution^32^. After extraction, most of the Si(3Al, 1Si) observed in the as-synthesized material shifts to Si(2Al, 2Si), indicating the incorporation of uncondensed Si in the tetrahedral framework of SAPO-37 in place of Al tetrahedral sites with a small amount of Si(0Al, 4Si) sites. The increase in intensity of T and Q sites of silicon environment resulted due to replacement of Al and P from the framework sites by organosilane moiety. The results are in accordance with the powder XRD pattern.

The ^13^C MAS NMR spectrum for as-prepared and extracted SAPO-37-DAS-0.16 samples exhibited five prominent signals at 10.3, 20.2, 36.5, 44.9 and 50.2 ppm, which can be assigned to various carbon environments present in the organosilane (N-[3-(trimethoxysilyl)propyl]ethylenediamine) species (Fig. 4B). The as-prepared materials showed addition signals with chemical shifts of 13.9 and 61.0 ppm, which could be assigned to carbon present in the template in different environments (TPA^+^)^33^. The peak at 58.5 ppm comes from the template TMA^+^ present in the sodalite cages of SAPO-37^33^. The peaks at 10.3 ppm are derived from methyl functionality present in both TPA^+^ and organosilane^32^. It is clear that the peaks corresponding to TMA^+^ disappeared in the extracted materials, and the intensity for TPA^+^ decreased considerably, which demonstrates the effective removal of template. The decrease in peak intensity with a chemical shift of 10.3 ppm could have resulted from a loss of template from super-cages of SAPO-37. The FT-IR, TGA and ^13^C MAS-NMR studies conclusively demonstrate the effective removal of template from the diaminosilane functionalized SAPO-37 framework. Hence, the extraction process helps in removal of template from the SAPO-37 framework.

The morphologies of diaminosilane functionalized SAPO-37 materials were studied using scanning electron microscopy (SEM). Pure SAPO-37 and SAPO-37-DAS obtained with different concentrations are shown in Figure S8. All the samples showed interpenetrated octahedral morphology typical of SAPO-37 structure with good crystallinity. The SEM micrographs clearly show the change in particle size and shape with varying diaminosilane concentrations.

**Figure 5:**
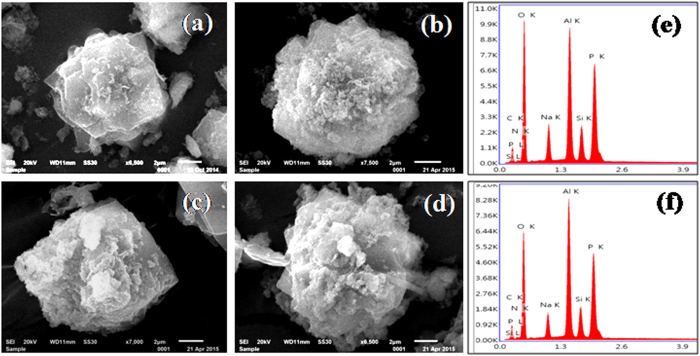
SEM images of (**a,c**) as-prepared diaminosilane functionalized SAPO-37 with organosilane (**a**) 0.16 and (**c**) 0.32; and (**b,d**) extracted diaminosilane functionalized SAPO-37 (**b**) 0.16 and (**d**) 0.32; and (**e,f**) EDAX for extracted (**e**) 0.16 and (**f**) 0.32 (Scale at bottom right side represents 2 μm).

An increase in external surface roughness with organosilane concentration without any aggregated phase was observed. This evidence supports the successful grafting of organosilane in the SAPO-37 framework. Figure 5 shows the SEM images for as-prepared and extracted SAPO-37-DAS-0.16 and SAPO-37-DAS-0.32 samples. In both cases, an increase in surface roughness was observed after template extraction with ethanolic sodium nitrate owing to presence of exposed surface diamine functionality. In addition, EDAX analysis of the extracted material showed relatively lower carbon content compared to as-prepared materials, further confirming qualitative removal of templates from the diaminosilane functionalized SAPO-37 framework.

The CHN elemental analysis of diaminosilane functionalized SAPO-37 prepared with different concentrations are shown in Table 1. The results show an increase in total N and C contents with an increase in organosilane concentration in synthesis gel. Importantly, in comparison to as-prepared materials, the SDA-extracted materials showed a drastic decrease in carbon content, once again demonstrating the effective removal of the template (TPA^+^ and TMA^+^) using novel ethanolic sodium nitrate extraction method. The results align with those of the TGA, FT-IR and NMR studies. Furthermore, the surface-exposed amine functionality on SAPO-37-DAS was estimated by argentometric titrations using 0.1 N AgNO_3_, and the results are summarized in Table 1. Based on the CHN analysis and argentometric titration, it was found that more than 90% of the diamine functional groups were accessible.

The textural properties of the ext-SAPO-37-DAS samples were studied using N_2_ sorption analysis, and the BET surface areas are shown in Table 1. The isotherms of extracted samples are typical of type I with a sharp uptake in relative pressure *(p/p*_*0*_) region less than 0.1 (as shown in Figure S9).With the increase in diaminosilane concentration decrease in surface area was observed owing to the presence of exposed organo-functional groups on the microporous SAPO-37 framework. The HK pore diameter was analyzed, although the method neglect the curvature on the forces of adsorption. The result showed decrease in pore width with increase in diaminosilane concentration. The result is in agreement with micropore volume derived from t-plot curve. The above fact clearly supports that the organo-functional group are present on the framework wall of SAPO-37.

The hydrothermal stability of materials was studied by treating as-prepared and extracted (SAPO-37-DAS-0.24) material with water in an autoclave for 6 h at 200 °C. The powder XRD pattern of as-prepared and extracted samples after hydrothermal treatments showed good crystallinity with retained faujasite planes (Figure S10). Hence the materials showed good hydrothermal stability which might have resulted due to presence of hydrophobic organosilane moiety in the framework.

**Figure 6:**
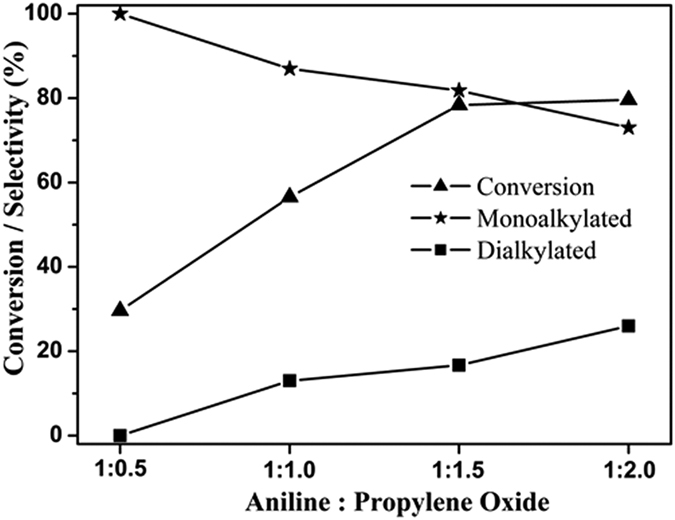
Catalytic activity of ext-SAPO-37-DAS-0.16 for ring opening of propylene oxide with aniline.

The catalytic activity of thoroughly characterized, ext-SAPO-37-DAS-0.16 was utilized for β-amino alcohol synthesis from aniline and thermally sensitive propylene oxide. The ring opening products of epoxide using aniline yield 1-(phenylamino) propan-2-ol and 2-(phenylamino) propan-1-ol, which are important intermediates in organic synthesis using a base catalyst^39^. The catalytic activity of diaminosilane functionalized SAPO-37 was studied in solvent-free conditions at different molar ratios of aniline and propylene oxide, as shown in Figure S11. The reaction was studied at room temperature in solvent-free conditions using ext-SAPO-37-DAS-0.16 at different molar ratios of propylene oxide to aniline (Fig. 6) ranging from 0.5 to 2.

The use of propylene oxide at low concentration results in moderate conversion with selective formation of mono-alkylated (mono-propylated) aniline as major product. The increase in propylene oxide molar ratio from 0.5 to 2 facilitates an increase in aniline conversion from 30 to 80% with the formation of a considerable amount of di-alklyated aniline products. The reaction does not proceed without catalyst and thus presence of surface diaminosilane functionality provides the required basicity and facilitates ring opening of propylene oxide. After the reaction, catalyst was washed with ethanol and dried at 90 °C, and the recyclability was studied. The catalyst retained about 80% of its original activity after three recycle experiments. Among the various functionalized catalysts (see Table 2) the one with ext-SAPO-37-DAS-0.16 showed better conversion and selectivity. The better conversion and selectivity can be explained based on textural properties (Table 1), where it has relatively high surface area and exposed active sites. The catalysts obtained using higher amine concentration showed slight decrease in catalytic activity could be due to the blocking of framework active sites by organosilane moiety, which was evident from surface area analysis.

The catalytic activity of ext-SAPO-37-DAS-0.16 was compared with conventional bases such as sodium hydroxide (NaOH), sodium carbonate (Na_2_CO_3_), magnesium oxide (MgO), magnesium-aluminium-hydrotalcite (MgAl-HT)^40^ under identical conditions and the results are summarized in Table 2. Among the various base catalyst studied SAPO-37-DAS-0.16 showed maximum conversion of about 78% and TOF with selective formation of mono-alkylated product. The catalytic conversion decreases in the order SAPO-37-DAS-0.16 > MgAl-HT > NaOH > MgO > Na_2_CO_3_.The uniform distribution of surface exposed active amine functionality on diaminosilane functionalized SAPO-37 framework facilitates the better catalytic activity. To understand the amount of external surface functional group, catalytic activity of as-prepared SAPO-37-DAS-0.16 was also studied under optimum conditions, which showed only about 50% conversion, which is about 65% of the total conversion. The above fact indicates the functionalized material possesses about 65% of active sites on the external surface and 35% in the super-cage of SAPO-37 framework. Thus, better catalytic activity was evident on template extracted samples. The above result emphasized that diaminosilane functionality is uniformly distributed inside the channel of SAPO-37 framework.

## Conclusions

In summary, a first report is provided for synthesis and effective removal of a structure directing agent (SDA)/template from diaminosilane functionalized microporous SAPO-37 molecular sieves by ion-exchange using ethanolic sodium nitrate solution. The *in-situ* preparation approach leads to homogeneously distributed organosilane within the framework walls without aggregation. The diaminosilane functionalized SAPO-37 structure remained intact with no phase change observed through pattern fitting using Le Bail method. The current ion-exchange extraction of SDA from as-prepared materials does not damage the structure, and the resultant materials showed promising catalytic activity for ring opening of epoxide with aniline to produce β-amino-alcohol. The developed materials showed better conversion compared with classical base catalysts.

## Experimental

Various chemicals such as pseudoboehmite (76% Al_2_O_3_; ACE, India), fumed silica (Aerosil-200) and N-[3-(trimethoxysilyl)propyl]ethylenediamine from Sigma Aldrich, *ortho*-phosphoric acid (H_3_PO_4_; 85%, Merck), 25 wt.% aqueous solution of tetramethylammonium hydroxide (TMAOH), 40 wt.% aqueous solution of tetrapropylammonium hydroxide (TPAOH) from Tritech Chemical, aniline (99%, CDH), propylene oxide (99%, Spectrochem) and deionized water were used. All chemicals were used as received without any further purification.

### *In-situ* preparation of diaminosilane-functionalized SAPO-37 (SAPO-37-DAS)

In a typical procedure, SAPO-37 gel was prepared using a molar gel composition of 1.0 (TPA)_2_O: 0.025 (TMA)_2_O: 1.0 Al_2_O_3_: 1.0 P_2_O_5_: 0.43 SiO_2_: 50 H_2_O^33^,^34^,^35^. About 5.25 g of pseudoboehmite and 9.17 g H_3_PO_4_ were dissolved in distilled water (solution 1) and stirred for 12 h. Then, a solution obtained by adding 1.03 g fumed silica into a mixture containing 40.5 g TPAOH and 0.73 g TMAOH solution was added and stirred for 2 h. The obtained gel was then transferred to a Teflon-lined autoclave and allowed to crystallize for 8 h at 200 °C. A specified amount of N-[3-(trimethoxysilyl)propyl]ethylenediamine (DAS) was added to the preformed gel, which was stirred for 1 h to obtain a homogeneous solution. The gel was further transferred to a Teflon autoclave and allowed to crystallize for an additional 16 h at 200 °C. The materials synthesized using 0.04, 0.08, 0.12, 0.16, 0.24, and 0.32 M of DAS with respect to the molar gel composition (Table S1) above are represented as SAPO-37-DAS-0.04, SAPO-37-DAS-0.08, SAPO-37-DAS-0.12, SAPO-37-DAS-0.16, SAPO-37-DAS-0.24, and SAPO-37-DAS-0.32, respectively.

### Templated removal procedure

The template present in the diaminosilane functionalized SAPO-37 was extracted using ion exchange method for the first time with an ethanolic solution of sodium nitrate (0.3 g in 50 ml ethanol; 0.04** **M). In a typical procedure, 1 g of the diaminosilane functionalized SAPO-37 sample was treated with (0.04** **M) ethanolic sodium nitrate solution. The mixture was refluxed at 80 °C for 6** **h; the procedure was repeated twice for complete removal of the template. After reflux, the samples were washed 2**–**3 times with ethanol, filtered and dried in an air oven at 90 °C for 12 h. After extraction the materials synthesized using 0.04, 0.08, 0.12, 0.16, 0.24, and 0.32 M of DAS with respect to the molar gel composition are represented as ext-SAPO-37-DAS-0.04, ext-SAPO-37-DAS-0.08, ext-SAPO-37-DAS-0.12, ext-SAPO-37-DAS-0.16, ext-SAPO-37-DAS-0.24, and ext-SAPO-37-DAS-0.32, respectively. For comparison, template present in diaminosilane functionalized SAPO-37 was also removed using conventional method such as HCl-ethanol, acetic acid-ethanol and NaCl-ethanol. In addition, the novel template extraction method was also extended to other microporous functionalized molecular sieves materials such as SAPO-40 and SAPO-5.

### Characterization

Powder X-Ray diffraction patterns of all the materials were collected on Brüker-D8 high resolution X-ray diffractometer with Cu-Kα radiation (λ = 1.5418 Å), between 2θ range of 4–40°, with a scan speed and step size of 0.5°/min and 0.02° respectively. FT-IR spectra were measured on Brücker optic model tensor 27 FT-IR spectrometer in 400–4000 cm^−1^ range using KBr pellets. Thermo gravimetric analysis (TGA) of materials was carried out on Perkin Elmer in air atmosphere with temperature ramp rate of 10°/min. The Solid-state NMR measurements were carried out on a Bruker AVANCE 400 narrow bore spectrometer equipped with a superconducting magnet of 9.4 T using a 4 mm double resonance magic angle spinning (MAS) probe operating at resonating frequencies of 79.4 and 100.62 MHz for ^29^Si and ^13^C respectively. The samples for ^29^Si and ^13^C were packed in 4 mm zirconia rotors and subjected to a spinning speed of 5 kHz; single pulse experiment with pulse duration of 4 μs and a relaxation delay time of 5 s were used for recording ^29^Si and ^13^C MAS NMR pattern with 2000 number of scans. The chemical shift values are expressed with respect to TMS for the ^29^Si and glycine ^13^C nucleus.The morphology of the materials was studied using scanning electron microscopy (SEM, JEOL, JSM 6610 LV with gold sputter coater JEC 300). Nitrogen adsorption/desorption isotherms were recorded on Micromeritics ASAP 2020, USA. The samples were degassed at 300 °C for 12–14 h under 0.1333 Pascal pressure and analysis was carried out at −196 °C prior to each analysis. The BET surface area was calculated in the relative pressure range (*p/p*_*0*_) 0.05-0.3, over the adsorption branch of the isotherm.

### Catalytic Studies

The diaminosilane functionalized SAPO-37 materials were utilized for ring opening of thermally sensitive propylene oxide using aniline. The reaction was carried out in round bottom flask connected with a reflux condenser. The products were analyzed in gas chromatography equipped with flame ionizing detector FID (Agilent 7890A Series) using HP-5 capillary column. For comparison, the catalytic activity of conventional bases such as NaOH, Na_2_CO_3_, MgO, magnesium-aluminium-hydrotalcite (MgAl-HT)^40^ were studied under identical conditions.

## Additional Information

**How to cite this article**: Yadav, R. *et al.* *In-situ* preparation of functionalized molecular sieve material and a methodology to remove template. *Sci. Rep.* **6**, 22813; doi: 10.1038/srep22813 (2016).

## Supplementary Material

Supplementary Information

## Figures and Tables

**Table 1:** Elemental analysis and textural properties for diamino-silane functionalized SAPO-37 materials.

SAPO-37-DAS-x x=	Elemental Analysis (CHN)				Total active sites mmol/g (CHN)	Accessible active sites mmol/g	Accessibility of accessible active site (%)	BET Surface area (cm^3^g^−1^)	Micropore	
	As-prepared (%)		Extracted (%)						t-plot volume (cm^3^g^−1^)	HK Pore width (nm)
	N	C	N	C						
0.04	1.35	16.05	1.03	5.71	0.95	0.25	26.3	202	0.113	1.55
0.08	1.56	15.25	1.18	6.55	1.22	0.34	27.8	211	0.076	1.24
0.12	1.96	14.30	1.62	7.12	1.43	1.11	77.7	220	0.074	1.24
0.16	2.43	14.29	1.92	7.94	1.52	1.46	96.1	219	0.066	1.24
0.24	2.64	19.03	2.64	8.74	1.71	1.67	97.6	188	0.058	1.24
0.32	2.90	16.00	3.21	10.30	2.07	2.05	99.0	146	–	–

**Table 2:** Comparison of catalytic activity of ext-SAPO-37-DAS with conventional base catalysts for ring opening of propylene oxide with aniline.^‡^

Catalyst	Conv. (%)	Selectivity (%)		TOF
		Mono-alkylated	Di-alkylated	
Ext-SAPO-37-DAS-0.04	54.5	86.8	13.2	3343
Ext-SAPO-37-DAS-0.08	61.1	85.1	14.9	2756
Ext-SAPO-37-DAS-0.12	68.2	84.6	15.4	942
Ext-SAPO-37-DAS-0.16	78.4	81.8	16.7	823
Ext-SAPO-37-DAS-0.24	71.9	80.2	19.8	660
Ext-SAPO-37-DAS-0.32	38.0	92.7	7.3	284
SAPO-37-DAS-0.16^¥^	49.6	89.3	10.6	521
*MgAl-HT^40^	59.4	84.8	15.2	607
^#^NaOH	47.1	90.9	9.4	481
MgO	4.7	100	–	49
Na_2_CO_3_	3.0	100	–	31

^‡^Reaction conditions: Aniline: Propylene ratio = 1: 1.5 (Temperature = room temperature, Solvent = neat; Substrate (aniline) : Catalyst ratio = 73. ^¥^ as-prepared sample; *MgAl-HT was synthesized as per reference 40; Catalyst amount used = 200 mg (based on basicity calculations). ^#^NaOH 0.1 ml of 1.5 M solution was used.
