# Supplementary material for: In-situ preparation of functionalized molecular sieve material and a methodology to remove template
Source: Sci Rep. 2016 Mar 10;6:22813. doi: 10.1038/srep22813 (PMC4785354; doi:10.1038/srep22813)
Supplement: Supplementary Information [file srep22813-s1.pdf]

In-situ preparation of functionalized molecular sieve material and a methodology to remove template

Rekha Yadav,[a] Maqsood Ahmed,[a] Arvind Kumar Singh[a] and Ayyamperumal Sakthivel*[a]

[a]Inorganic Materials and Catalysis Laboratory, Department of Chemistry

University of Delhi, Delhi 110 007, India

Email: sakthiveldu@gmail.com / asakthivel@chemistry.du.ac.in

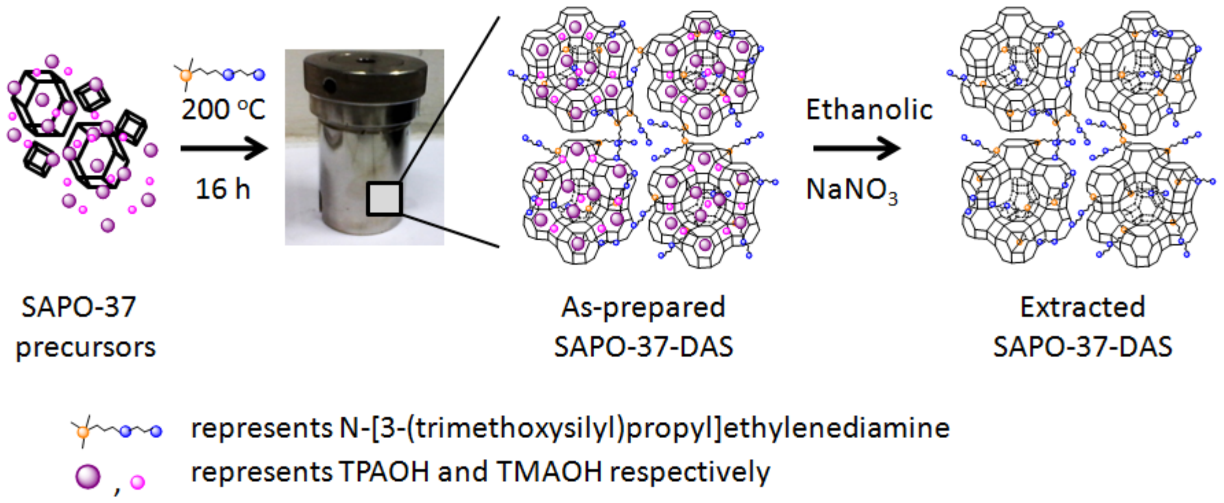

FigureS1. Schematic representation of *in-situ* synthesis of diaminosilane functionalized SAPO-37 and their template removal by ion-exchange.

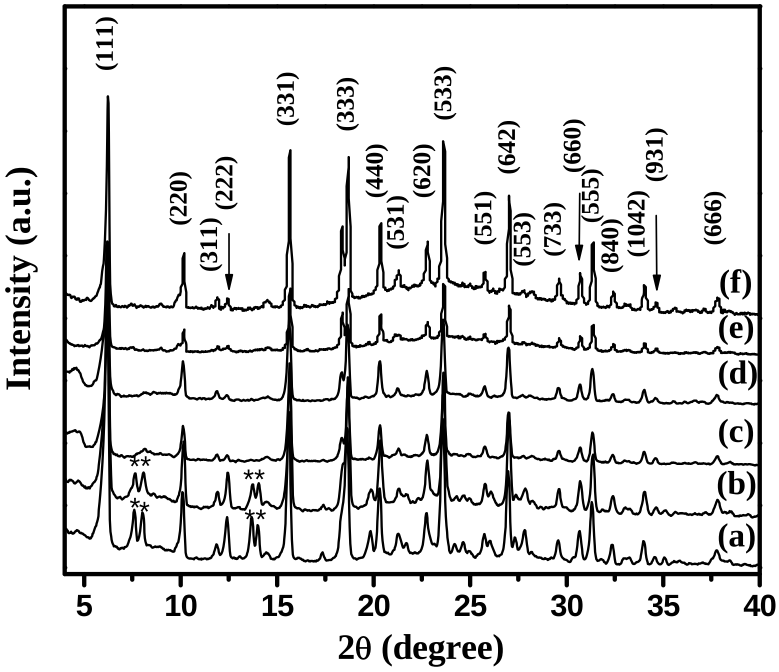

Figure S2. Powder XRD pattern of as-prepared diaminosilane functionalized SAPO-37 with organosilane concentration (a) 0.04 (b) 0.08 (c) 0.12 (d) 0.16 (e) 0.24 and (f) 0.32.

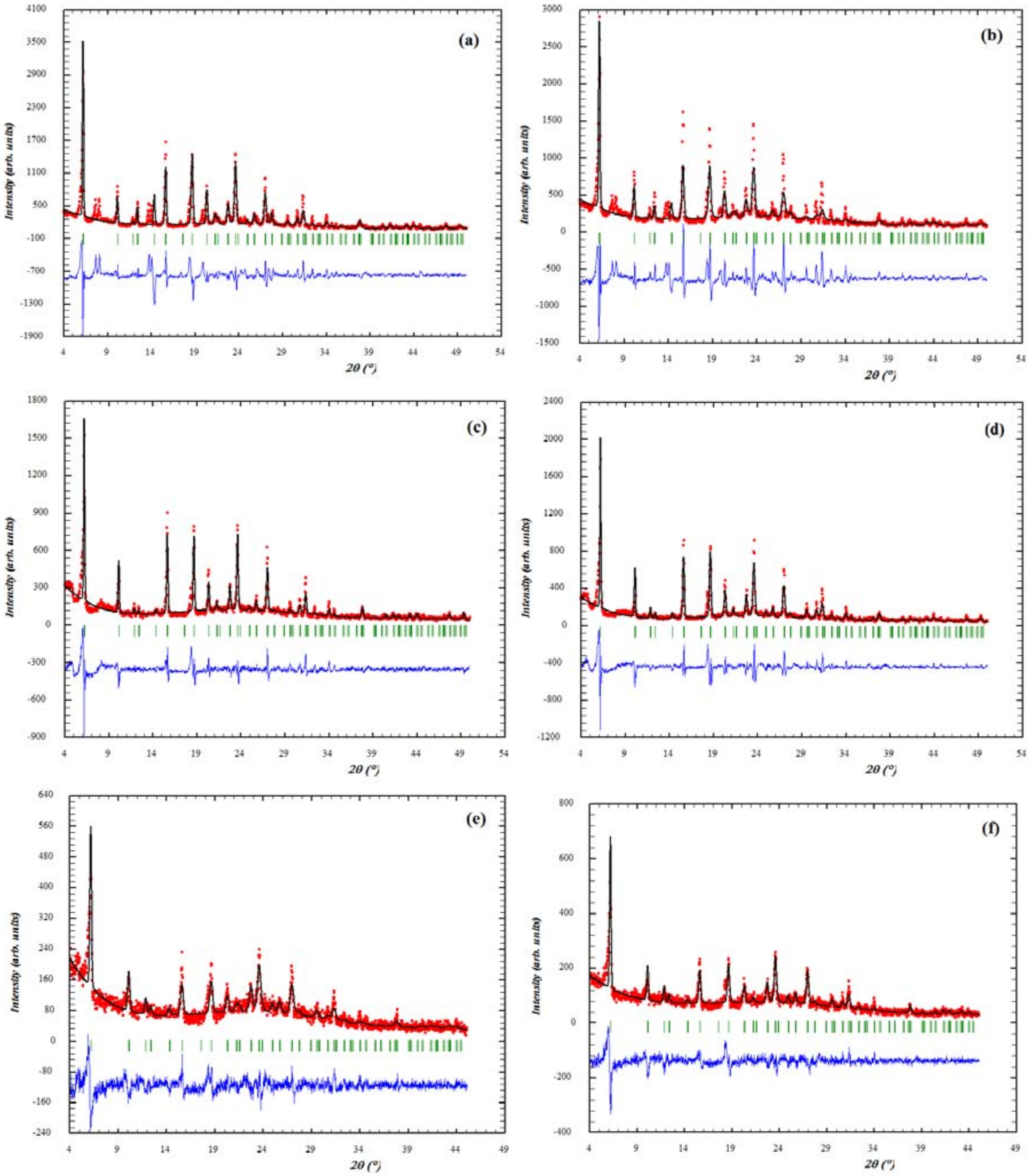

Figure S3. Powder XRD pattern of diaminosilane functionalized SAPO-37 with different organosilane concentrations (a) 0.04 (b) 0.08 (c) 0.12 (d) 0.16 (e) 0.24 and (f) 0.32 and its fitting by Le Bail method. Vertical ticks correspond to line indexing (green color) of SAPO-37(FAU phase). Difference plots between calculated (black line) andexperimental points (red dots) are shown at the bottom (blue color) of each pattern.

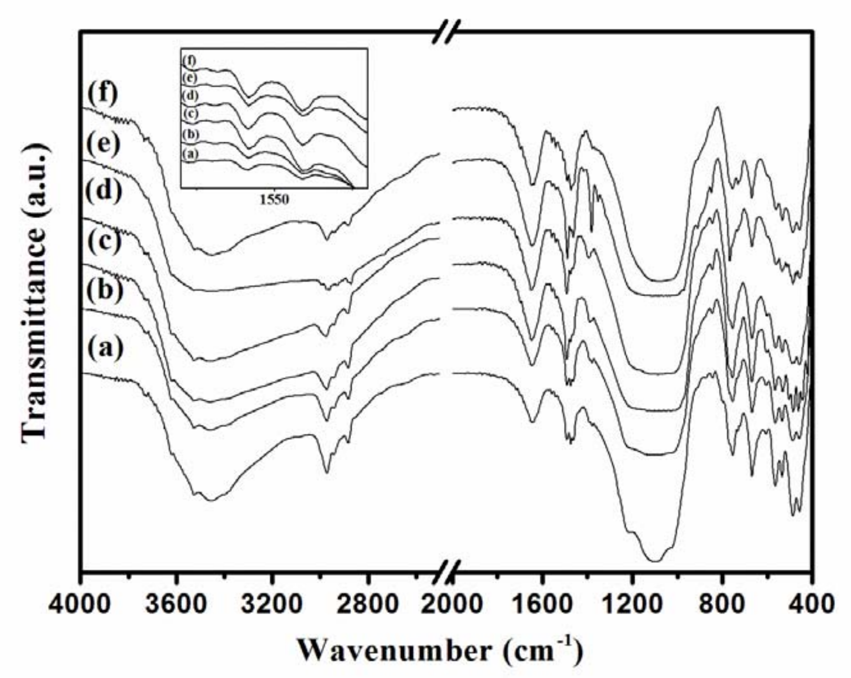

Figure S4. FT-IR spectra of as-prepared diaminosilane functionalized SAPO-37 with organosilane concentration (a) 0.04 (b) 0.08 (c) 0.12 (d) 0.16 (e) 0.24 and (f) 0.32.

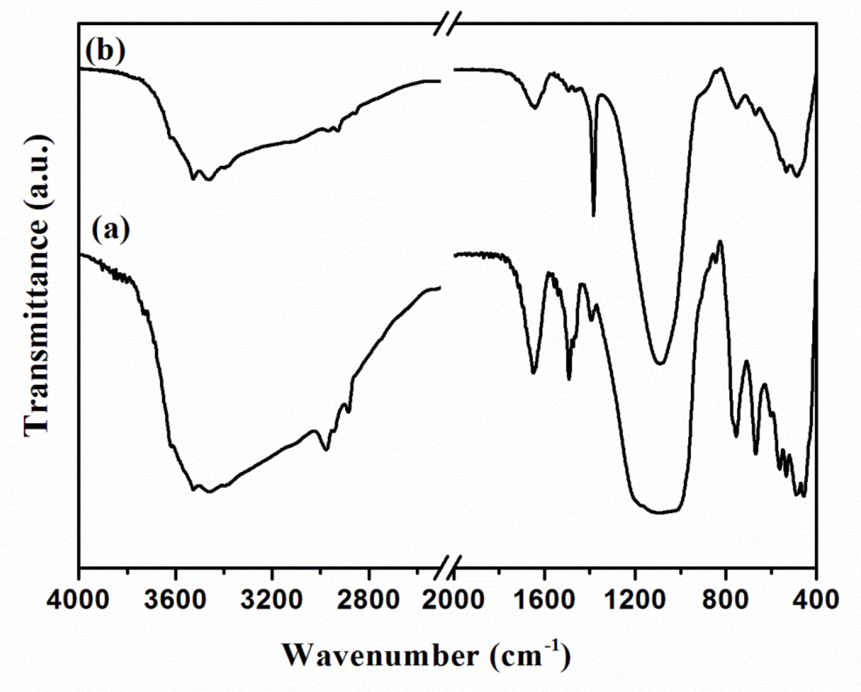

Figure S5. FT-IR spectra of diaminosilane functionalized SAPO-37-DAS-0.16 (a) as-prepared (b) extracted.

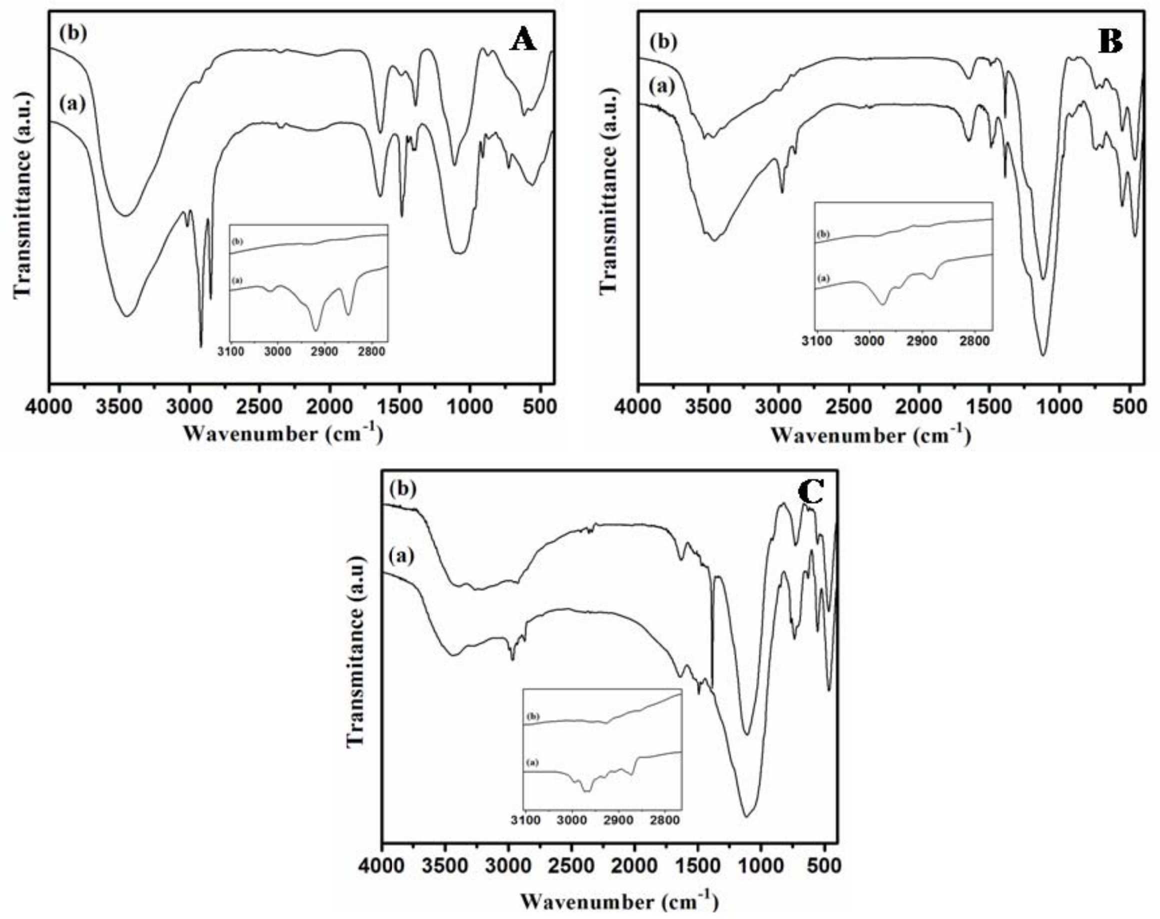

Figure S6. FT-IR of (A) Mesoporous Silicoaluminophosphate (MESO-SAPO-37)(B) functionalized SAPO-40 (C) functionalized SAPO-5 where (a) As-prepared (b) Solvent extracted.

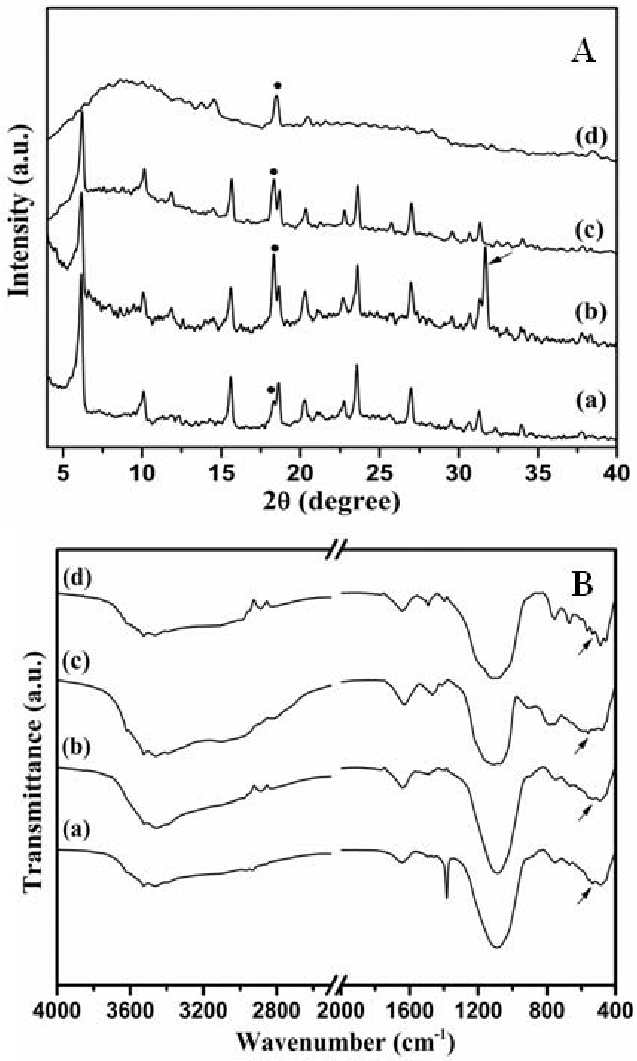

Figure S7. Powder XRD pattern (A) and FT-IR spectra (B) for SAPO-37-DAS-0.16 extracted with (a) ethanolic $NaNO_3$, (b) ethanolic NaCl, (c) ethanolic acetic acid (d) ethanolic HCl (• represents alumina impurity).

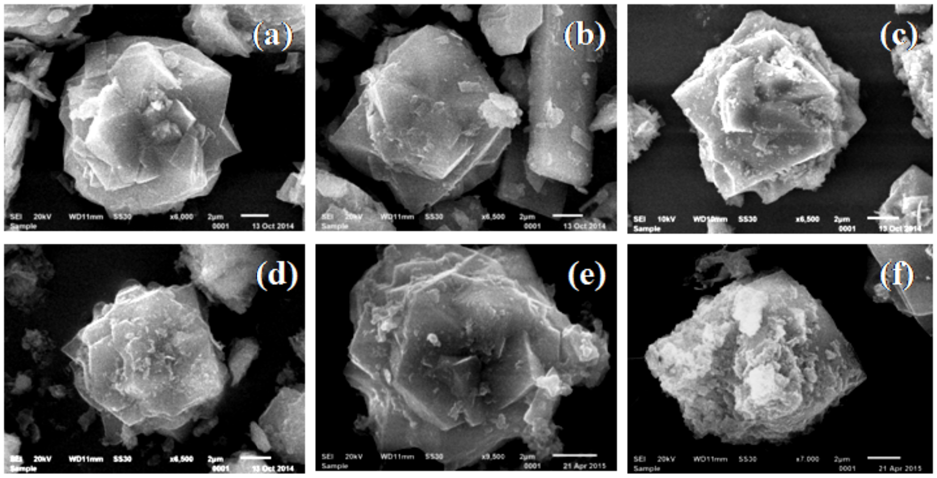

Figure S8.SEM images of as-prepared SAPO-37 (a) without organosilane and with organosilane (b) 0.04 (c) 0.08 (d) 0.16 (e) 0.24 and (f) 0.32 (Scale at bottom right side represents 2 μm).

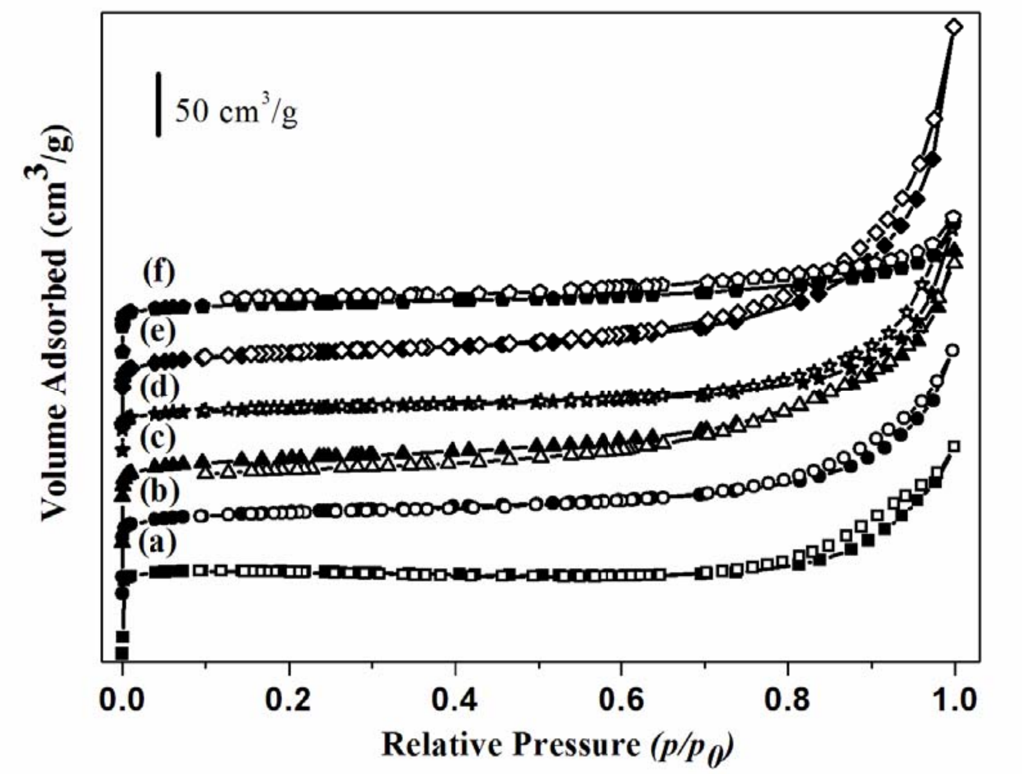

Figure S9. N_2 sorption of extracted diaminosilane functionalized SAPO-37 with organosilane (a) 0.04 (b) 0.08 (c) 0.12 (d) 0.16 (e) 0.24 and (f) 0.32.

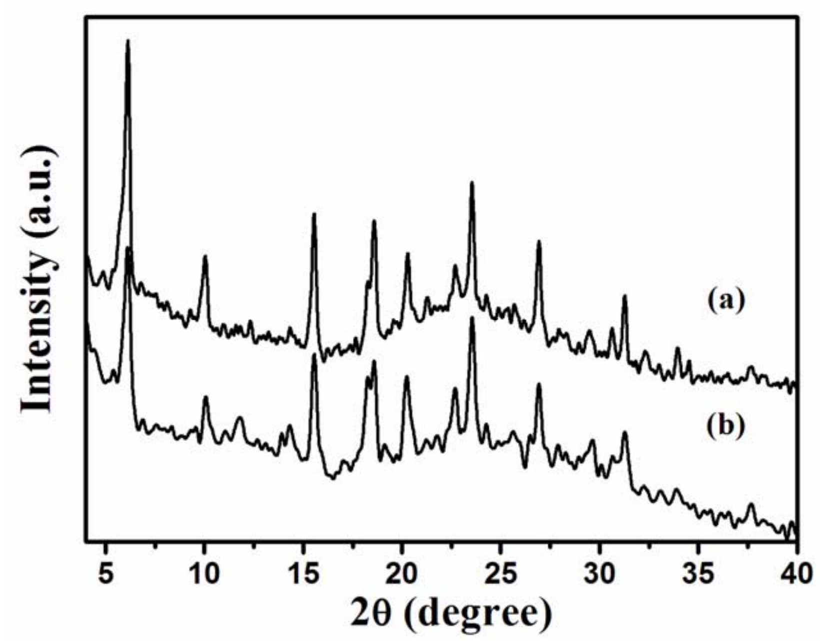

Figure S10. Powder XRD pattern for (a) as-prepared SAPO-37-DAS-0.24 6 h VPT at 200 ^{o}C (b) ext-SAPO-37-DAS-0.24 6 h in VPT at 200 ^{o}C. (In VPT treatment 200 mg of material was treated with water vapor in Teflon beaker with 30 % of water in base of an autoclave)

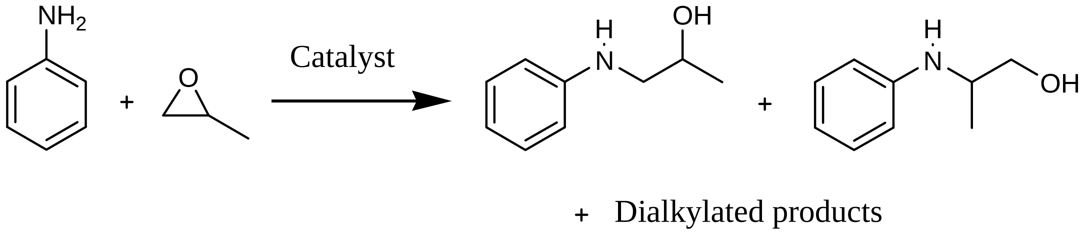

Figure S11. Schematic representation of ring opening of propylene oxide with aniline.

Table Captions

Table S1. Synthesis gel composition of diaminosilane functionalized materials.

Sample Code	Gel composition (molar ratio)	Silane Concentra-tion (M)	Total Si (M)
SAPO-37-DAS-0.04	1.0 $(TPA)_2O$: 0.025 $(TMA)_2O$: 1.0 Al_2O_3: 1.0 P_2O_5: 0.43 SiO_2: 0.04DAS: 50 H_2O	0.04	0.442
SAPO-37-DAS-0.08	1.0 $(TPA)_2O$: 0.025 $(TMA)_2O$: 1.0 Al_2O_3: 1.0 P_2O_5: 0.43 SiO_2: 0.08DAS: 50 H_2O	0.08	0.455
SAPO-37-DAS-0.12	1.0 $(TPA)_2O$: 0.025 $(TMA)_2O$: 1.0 Al_2O_3: 1.0 P_2O_5: 0.43 SiO_2: 0.12DAS: 50 H_2O	0.12	0.468
SAPO-37-DAS-0.16	1.0 $(TPA)_2O$: 0.025 $(TMA)_2O$: 1.0 Al_2O_3: 1.0 P_2O_5: 0.43 SiO_2: 0.16DAS: 50 H_2O	0.16	0.480
SAPO-37-DAS-0.24	1.0 $(TPA)_2O$: 0.025 $(TMA)_2O$: 1.0 Al_2O_3: 1.0 P_2O_5: 0.43 SiO_2: 0.24DAS: 50 H_2O	0.24	0.506
SAPO-37-DAS-0.32	1.0 $(TPA)_2O$: 0.025 $(TMA)_2O$: 1.0 Al_2O_3: 1.0 P_2O_5: 0.43 SiO_2: 0.32DAS: 50 H_2O	0.32	0.531

Table S2. Calculated cell parameters based on Le Bail pattern fitting method of as-prepared and extracted samples.

SAPO-37-DAS-x x=	Le Bail method	
	As-prepared (a=b=c) (Å)	Extracted (a=b=c) (Å)
0.04	24.79	24.68
0.08	24.68	24.68
0.12	24.70	24.71
0.16	24.74	24.70
0.24	24.70	24.70
0.32	24.73	24.71
